# Management of the Knee Extension Deficit after Acute Trauma

**DOI:** 10.1155/2018/5906109

**Published:** 2018-12-09

**Authors:** Thiago Alvim do Amaral, David Sadigursky

**Affiliations:** ^1^Hospital Manoel Victorino, Clínica Ortoped, Salvador, Bahia, Brazil; ^2^Hospital Manoel Victorino, Salvador, Bahia, Brazil

## Abstract

A 25-year-old man initially presented with right knee extension deficit after an acute trauma, caused by a condition known as arthrogenic muscle inhibition. This should not be confused with a mechanical block caused by intra-articular pathology. The loss of knee extension, even if minimal, is disabling and leads to worse results after knee surgical treatment. Therefore, it is necessary to recognize and diagnose arthrogenic muscle inhibition to ensure the best treatment for patients with this condition. In this case report, the patient was managed with a rehabilitation technique resulting in an effective functional gain of the quadriceps and full restoration of knee extension.

## 1. Introduction

Knee extension deficit after acute trauma is a painful and debilitating orthopedic condition. It usually results from a mechanical block caused by an intra-articular pathology [1]. However, more recently, some reports have suggested that the extension deficit may be due to a process known as arthrogenic muscle inhibition (AMI) [2].

AMI is a reflex response occurring after a knee joint injury that causes a pseudolocked knee. The exact pathophysiology is not clearly understood but is believed that some factors such as swelling and inflammation caused by an acute knee trauma can lead to changes in the discharges of sensory receptors. These mechanisms may alter afferent signals sent to the nervous system, thereby causing an involuntary hamstring contracture with a selective quadricipital shutdown [2].

Our report presents the case of a 25-year-old male who suffered a torsional trauma to the left knee. He was initially referred to our service with an indication to perform emergency surgery due to suspicion of mechanical blockage caused by a meniscal rupture evidenced through MRI. After evaluation, we realized that it was, in fact, a “pseudo locking of the knee.”

Although arthrogenic muscle inhibition is related to a knee extension deficit resulting from an injury, there are few studies about this subject; therefore, it may go underdiagnosed in the orthopedic field. Suspected AMI patients should not be submitted to surgical procedures without a prior attempt to go through conservative procedures aiming to reach a complete knee extension. Surgical procedures can lead to disastrous results in this scenario [3, 4]. The purpose of this report is to highlight the importance of correct diagnosis and adequate management of AMI to avoid long-term disability caused by this dysfunction.

## 2. Case Report

**Figure 1:**
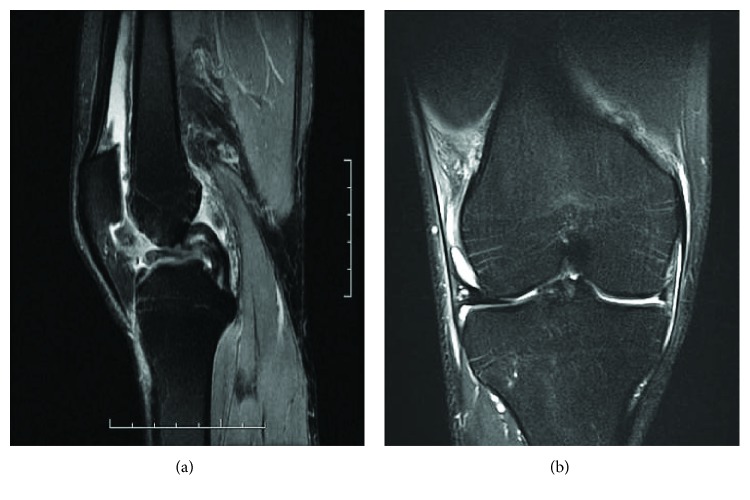
Magnetic resonance imaging in T2 of the knee with sagittal and coronal sections demonstrating the bucket handle tear of the medial meniscus.

A 25-year-old male patient, victim of torsional trauma in the right knee during a soccer match, was brought to the emergency department with a locked knee. At the time of the injury, the patient felt a pop and immediately after, he could not move his knee. Plain radiographs of the knee showed no signs of acute osteoarticular lesions. Subsequently, magnetic resonance imaging identified a bucket handle tear in the medial meniscus (Figure 1). The patient was referred to the orthopedic trauma service for emergency surgical treatment.

**Figure 2:**
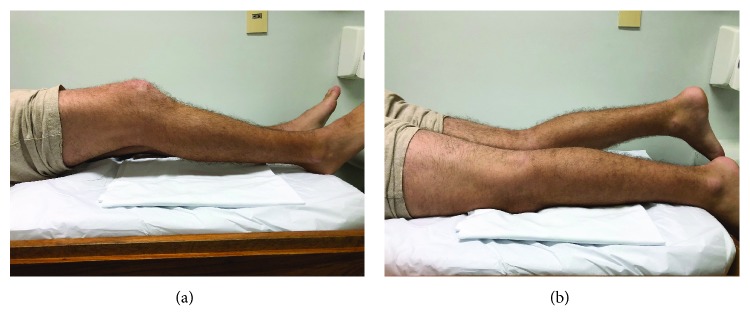
Photograph demonstrating the initial extension deficit of the right knee.

**Figure 3:**
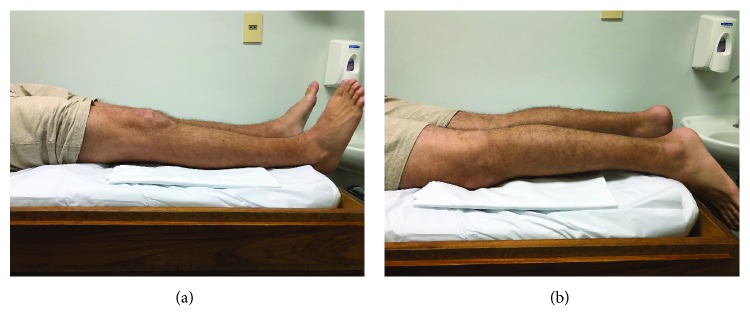
Photograph showing the gain of the complete extension after the completion of the proposed exercises.

One week after the injury, the patient sought our service. Physical examination revealed the following findings: joint effusion, 30 degrees of flexion, and an inability to actively contract the quadriceps. In the prone position, an important contracture of the hamstring muscle group was observed when compared to the contralateral side (Figure 2). Based on the patient's history and physical examination, we suspected that the extension deficit resulted from arthrogenic muscle inhibition. A set of exercises was performed, using the technique described by Delaloye et al. [5], which usually results in full restoration of the knee extension: the patient was initially placed in prone position, with the feet off the stretcher, and asked to perform active contraction of the hamstrings. The contraction should be held for 2 to 3 seconds. Between contractions, the patient was asked to completely relax the hamstrings. This sequence of exercises was performed repeatedly until fatigue was observed, at which point, a complete hamstring relaxation had occurred. Once full passive extension was recovered, the patient was placed in dorsal decubitus, with the trunk elevated to 90 degrees and with the lower limb in extension. In this position, the patient was instructed to perform isometric contractions of the quadriceps (vastus medialis) until he succeeded in performing an adequate contraction. The patient in our report presented complete extension after approximately 7 min of the resisted exercises (Figure 3).

## 3. Discussion

Knee extension deficit is a relatively common presentation in patients suffering from acute knee trauma. When treating a patient with this condition, it is important to differentiate between two distinct entities: (i) a mechanical block caused by displacement of an intra-articular structure (meniscus, loose bodies) or (ii) a “pseudolocked knee” due to a process known as arthrogenic muscle inhibition [6].

Proper physical examination is an important step in the search for the correct diagnosis. In patients with arthrogenic muscle inhibition, the pattern of contraction of the quadriceps muscle should be observed. Usually the rectus femoris maintains its ability to contract and thereby flex the hip, while the vastus medialis is unable to contract and thereby extend the knee. An easy way to check for proper muscle contraction is through palpation of the patella. During each quadriceps contraction, the patella should migrate proximally; however, in patients with knee extension deficit due to arthrogenic muscle inhibition, only the rectus femoris contracts. The thigh muscles contract, but no movement of the patella occurs. Then, with the patient in prone position, palpation of the involved side compared to the contralateral side allows to determine the presence of muscular contracture. If the hamstring contracture is not present, a mechanical block caused by the displacement of an intra-articular structure should be considered [5].

This case is emblematic because it demonstrates the fact that a displaced bucket handle tear of the meniscus evidenced by MRI does not define whether the extension deficit is actually due to a mechanical block. Shakespeare and Rigby [6] evaluated 272 patients with a bucket loop meniscal lesion, but only 43% of them presented a knee block at the time of evaluation. Allum and Jones [1] prospectively investigated 50 patients who presented with a blocked knee and observed that only 16% of them remained blocked after anesthetic induction. They concluded that the knee extension deficit observed in most patients was due to hamstring muscle spasms secondary to arthrogenic muscle inhibition.

Some promising interventions to mitigate the effects of arthrogenic muscle inhibition include cryotherapy, transcutaneous electrical nerve stimulation (TENS), and neuromuscular electrical stimulation; however, these treatments require additional equipment, are time-consuming, and produce varied results [2, 6]. The method described by Delaloye et al. [5] is simple, with a small, fast, and effective learning curve and restores complete knee extension and quadriceps muscle activation. The described exercise set specifically targets arthrogenic muscle inhibition, reduces the influence of spinal hyperreflexia and fatigue of the hamstrings and combats cortical neuroplasticity through the repetition of quadriceps activation exercises [5].

Orthopedic surgeons deal daily in their clinical practice with cases of locked knee. Therefore, it is important for orthopedists to acquaint themselves with the prevalence and clinical effects of arthrogenic muscle inhibition in addition to its effective treatment in order to devise strategies to overcome this impairment.
